# Medical History of Circulatory Diseases and Colorectal Cancer Death in the JACC Study

**DOI:** 10.2188/jea.15.S168

**Published:** 2005-08-18

**Authors:** Yoshiyuki Watanabe, Kotaro Ozasa, Yoshinori Ito, Koji Suzuki, Masayo Kojima, Sadao Suzuki, Shinkan Tokudome, Koji Tamakoshi, Hideaki Toyoshima, Miyuki Kawado, Shuji Hashimoto, Norihiko Hayakawa, Kenji Wakai, Akiko Tamakoshi

**Affiliations:** 1 Department of Epidemiology for Community Health and Medicine, Kyoto Prefectural University of Medicine Graduate School of Medical Science.; 2 Department of Public Health, Fujita Health University School of Health Sciences.; 3 Department of Health Promotion and Preventive Medicine, Nagoya City University Graduate School of Medical Sciences.; 4 Department of Public Health/Health Information Dynamics, Nagoya University Graduate School of Medicine.; 5 Department of Hygiene, Fujita Health University School of Medicine.; 6 Department of Epidemiology, Research Institute for Radiation Biology and Medicine, Hiroshima University.; 7 Division of Epidemiology and Prevention, Aichi Cancer Center Research Institute.; 8 Department of Preventive Medicine/Biostatistics and Medical Decision Making, Nagoya University Graduate School of Medicine.

**Keywords:** Medical history, Hypertension, Myocardial Infarction, Colorectal Neoplasms, Cohor Studies, Epidemiology

## Abstract

BACKGROUND: Host factors expressed by individual past medical history of hypertension, stroke, and myocardial infarction may have a relationship with colorectal cancer.

METHODS: As part of the Japan Collaborative Cohort Study (JACC Study) for the Evaluation of Cancer Risk sponsored by the Ministry of Education, Science, Sports and Culture of Japan (Monbusho), we conducted a follow-up study of 110,792 Japanese inhabitants aged 40-79 years to reveal the relationship of past medical history of hypertension, stroke, and myocardial infarction at the baseline in 1988-1990 with colorectal cancer death for about 10 years up to the end of 1999.

RESULTS: Past medical history of hypertension associated with an increased risk of female rectal cancer when analyzing all cancer cases with adjustment for age, body mass index, and exercise (hazard ratio [HR] = 1.97, 95% confidence interval [CI]; 1.13-3.43). Past medical history of myocardial infarction was also an increased risk for female rectal cancer (HR = 3.05, 95% CI; 1.28-7.28). Females who had a medical history of stroke had increased risk of rectal cancer without statistical significance.

CONCLUSION: There was a positive association of past medical history of hypertension and myocardial infarction and an increased risk of rectal cancer in women.

We reported a positive association between past medical history of hypertension and colorectal cancer in a case-control study in early 1980,^1^ although it was not related to colorectal cancer in a recent case-control study in Italy.^2^ Patients with hypertension, stroke and myocardial infarction may have similar lifestyle factors, such as high intake of dietary fat, less frequent physical exercise and obesity, to colorectal cancer cases. These common risk factors may contribute to the positive association between the above circulatory diseases and colorectal cancer.

Therefore, we conducted a cohort study to reveal the relationship in the Japan Collaborative Cohort Study (JACC Study) for the Evaluation of Cancer Risk sponsored by the Ministry of Education, Science, Sports and Culture of Japan (Monbusho).

## METHODS

The study population, procedures for conducting the baseline survey using a self-administered questionnaire, and follow-up methods in the JACC Study have been described previously.^3^^,^^4^ Briefly, the study population was 110,792 Japanese inhabitants aged 40-79 years at 45 study areas in 1988-1990. Subjects completed a self-administered questionnaire including past medical history of hypertension, stroke, and myocardial infarction. A response to the medical history questions was selected from four alternatives such as “have never suffered from the disease”, “have suffered from the disease with present treatment”, “have suffered from the disease with treatment”, and “have suffered from the disease without treatment”. Therefore, subjects with positive medical history of hypertension, stroke and myocardial infarction were defined as those who had suffered from hypertension, stroke and myocardial infarction, respectively, irrespective of past and present treatment.

The follow-up survey was conducted using population registries in local municipalities to determine the vital and residential status of the cohort in each area. All subjects that moved out of the study areas were treated as censored subjects. All deaths that occurred in the cohort were ascertained by death certificates from local public health centers in the study areas with the authorities’ permission from the Director-General of the Prime Minister’s Office (Ministry of Public Management, Home Affairs, Post, and Telecommunications). The causes of death were coded according to the International Statistical Classification of Diseases and Related Health Problems, 10th Revision^5^ by verifying computer-stored data in the Ministry of Health, Labour, and Welfare with permission. A diagnosis of colon cancer was defined by code C18, while rectal cancer was C19 and C20, in the above classification.^5^

The risk of colorectal cancer was evaluated by hazard ratios (HRs) and 95% confidence intervals (CIs) estimated by the Cox proportional hazards model. Sex-specific HRs were computed after adjustment for age in all colorectal cancer cases and cases except for those who died within the first two years of the follow-up period. Sex-specific HRs were also computed after adjustment for age, body mass index (BMI) (25+, 20-25, -20 kg/m^2^, and no answer) and exercise (5+, 3-4, 1-2 hours per week, seldom, and no answer) for all colorectal cancer cases and cases except for those who had died within the first two years of the follow-up period. Body mass index (BMI) was calculated as weight (kg) divided by height (m) squared provided in the self-administered questionnaire at the baseline.

Individual written or oral consent, or consent from community representatives, was obtained, or a poster notification/opting-out system was applied.^3^^,^^4^ The Ethical Boards of Nagoya University School of Medicine and Fujita Health University approved this study.

## RESULTS

When the data were analyzed with adjustment for age only, past medical history of hypertension increased the risk of female rectal cancer for all cancer cases (HR = 1.78, 95% CI; 1.02-3.11) and for cases except for those died within the first two years of the follow-up period (HR = 1.85, 95% CI; 1.03-3.33). Past medical history of stroke and myocardial infarction had a positive association with female rectal cancer, although there was no statistical significance. Past medical history of hypertension, stroke, and myocardial infarction also showed a positive association with male colon cancer, although none were not statistically significant. There were no other significant relationships among past medical history of hypertension, stroke, and myocardial infarction with colorectal cancer death by sex or site of cancer (colon/rectum).

Sex-specific HRs and 95% CIs of colon cancer and rectal cancer adjusted by age, BMI and exercise are shown in Table 1. Past medical history of hypertension increased the risk of female rectal cancer when analyzing all cancer cases (HR = 1.97, 95% CI; 1.13-3.43) and cases except for those died within the first two years of the follow-up period (HR = 2.08, 95% CI; 1.16-3.73). Past medical history of myocardial infarction also increased the risk of female rectal cancer when analyzing all cancer cases (HR = 3.05, 95% CI; 1.28-7.28) and cases except for those who died within the first two years of the follow-up period (HR = 3.54, 95% CI; 1.47-8.53). There were no other significant results.

**Table 1.** Sex-specific hazard ratio (HR) and 95% confidence interval (CI) of past history of hypertension, stroke, and myocardial infarction for colon and rectal cancer mortality adjusted for age, body mass index and exercise.

	All causes				All causes except for those died within the first two years of the follow-up		
	
	Person-years	No. of Cases	Adjusted HR* (95% CI)		Person-years	No. of Cases	Adjusted HR* (95% CI)
				Colon cancer			

Males							
Hypertension	404,090	124	1.01 (0.68 - 1.51)		322,051	110	0.99 (0.64 - 1.52)
Stroke	393,120	122	1.64 (0.72 - 3.75)		313,469	108	1.96 (0.85 - 4.50)
Myocardial infarction	394,168	120	1.25 (0.58 - 2.69)		314,282	106	1.49 (0.69 - 3.23)
Females							
Hypertension	573,184	130	0.77 (0.52 - 1.13)		459,204	121	0.82 (0.55 - 1.23)
Stroke	552,990	124	0.96 (0.24 - 3.92)		443,152	116	1.05 (0.26 - 4.23)
Myocardial infarction	555,667	128	1.08 (0.50 - 2.33)		445,254	120	1.17 (0.54 - 2.52)

				Rectal cancer			

Males							
Hypertension	404,090	109	0.98 (0.63 - 1.51)		322,051	104	0.92 (0.59 - 1.45)
Stroke	393,120	104	0.33 (0.05 - 2.34)		313,469	100	0.35 (0.05 - 2.49)
Myocardial infarction	394,168	105	0.41 (0.10 - 1.67)		314,282	101	0.44 (0.11 - 1.79)
Females							
Hypertension	573,184	54	1.97 (1.13 - 3.43)		459,204	48	2.08 (1.16 - 3.73)
Stroke	552,990	47	2.99 (0.72-12.45)		443,152	41	3.45 (0.83-14.43)
Myocardial infarction	555,667	49	3.05 (1.28 - 7.28)		445,254	43	3.54 (1.47 - 8.53)

* : Adjusted hazard ratio for all cases by age, body mass index, and exercise.

## DISCUSSION

There are no consistent epidemiologic reports on the relationship between past medical history of hypertension and colorectal cancer.^1^^,^^2^ The present study showed a positive association between past medical history of hypertension and female rectal cancer. Females who had a medical history of hypertension had an increased risk of rectal cancer. This result supports the idea of the presence of common risk factors between colorectal cancer and hypertension. Obesity and physical inactivity are risk factors for hypertension.^6^ Obesity^7^^,^^8^ and physical inactivity^8^^,^^9^ are also risk factors for colorectal cancer. However, the risk of rectal cancer still existed when BMI and exercise were adjusted. Therefore, there may be common risk factors other than BMI and exercise involved in the link between hypertension and rectal cancer deaths. Females who had a medical history of myocardial infarction also had increased risk of rectal cancer when BMI and exercise were adjusted. Females who had a medical history of stroke had increased risk of rectal cancer without statistical significance. This may be accounted for by hypertension, as myocardial infarction and stroke are both closely related to hypertension.

**Figure 1.**
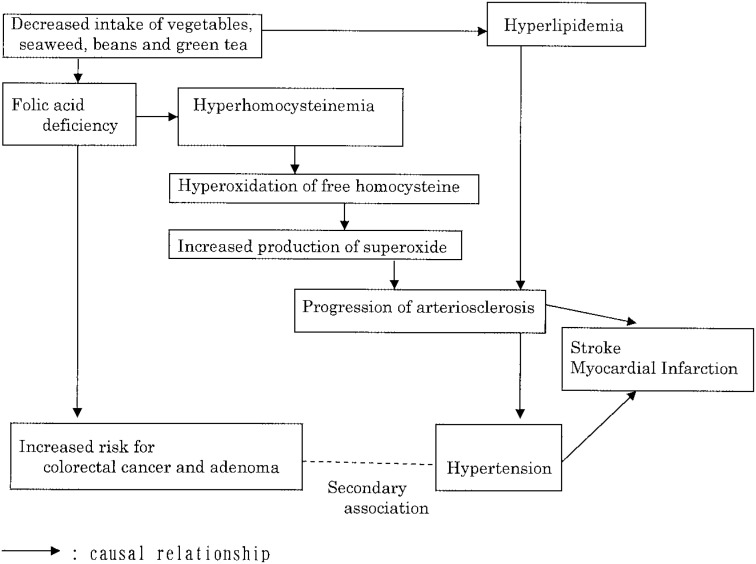
Hypothetical correlation among hypertension, stroke, and myocardial infarction and colorectal cancer (modified version of the original figure^10^)

There is a hypothetical correlation between circulatory diseases and colorectal cancer, shown in Figure 1, from the viewpoint of folic acid metabolism.^10^ We proposed this hypothesis because the average value of plasma homocysteine of patients with colorectal cancer was significantly higher than of healthy controls in a clinical case-control study.^10^ Except for folic acid deficiency, oxidative stress may contribute to the relationship. We have already reported that higher levels of serum oxidized low-density lipoprotein, which is believed to play a role in the development and progression of atherosclerosis,^11^ were associated with risk of colorectal cancer as part of the JACC Study.^12^ Therefore, our findings that past medical history of hypertension and myocardial infarction increased the risk of female rectal cancer could be a secondary association due to folic acid deficiency and /or oxidative stress.

There is a major limitation in interpreting the present results. As we conducted a cohort study, some of the subjects who did not have a medical history of hypertension at the time of the baseline questionnaire survey may have developed hypertension and then subsequently colorectal cancer during the follow-up period. Therefore, the relationship between past medical history and colorectal cancer death might have been underestimated.

There were no consistent results by sex or site of cancer (colon/rectum) in our study. These differences should be carefully addressed by considering factors other than folic acid deficiency and /or oxidative stress, such as hormonal background.

## MEMBER LIST OF THE JACC STUDY GROUP

The present investigators involved, with the co-authorship of this paper, in the JACC Study and their affiliations are as follows: Dr. Akiko Tamakoshi (present chairman of the study group), Nagoya University Graduate School of Medicine; Dr. Mitsuru Mori, Sapporo Medical University School of Medicine; Dr. Yutaka Motohashi, Akita University School of Medicine; Dr. Ichiro Tsuji, Tohoku University Graduate School of Medicine; Dr. Yosikazu Nakamura, Jichi Medical School; Dr. Hiroyasu Iso, Institute of Community Medicine, University of Tsukuba; Dr. Haruo Mikami, Chiba Cancer Center; Dr. Yutaka Inaba, Juntendo University School of Medicine; Dr. Yoshiharu Hoshiyama, Showa University School of Medicine; Dr. Hiroshi Suzuki, Niigata University School of Medicine; Dr. Hiroyuki Shimizu, Gifu University School of Medicine; Dr. Hideaki Toyoshima, Nagoya University Graduate School of Medicine; Dr. Shinkan Tokudome, Nagoya City University Graduate School of Medical Science; Dr. Yoshinori Ito, Fujita Health University School of Health Sciences; Dr. Shuji Hashimoto, Fujita Health University School of Medicine; Dr. Shogo Kikuchi, Aichi Medical University School of Medicine; Dr. Akio Koizumi, Graduate School of Medicine and Faculty of Medicine, Kyoto University; Dr. Takashi Kawamura, Kyoto University Center for Student Health; Dr. Yoshiyuki Watanabe, Kyoto Prefectural University of Medicine Graduate School of Medical Science; Dr. Tsuneharu Miki, Kyoto Prefectural University of Medicine Graduate School of Medical Science; Dr. Chigusa Date, Faculty of Human Environmental Sciences, Mukogawa Women’s University ; Dr. Kiyomi Sakata, Wakayama Medical University; Dr. Takayuki Nose, Tottori University Faculty of Medicine; Dr. Norihiko Hayakawa, Research Institute for Radiation Biology and Medicine, Hiroshima University; Dr. Takesumi Yoshimura, Institute of Industrial Ecological Sciences, University of Occupational and Environmental Health, Japan; Dr. Akira Shibata, Kurume University School of Medicine; Dr. Naoyuki Okamoto, Kanagawa Cancer Center; Dr. Hideo Shio, Moriyama Municipal Hospital; Dr. Yoshiyuki Ohno, Asahi Rosai Hospital; Dr. Tomoyuki Kitagawa, Cancer Institute of the Japanese Foundation for Cancer Research; Dr. Toshio Kuroki, Gifu University; and Dr. Kazuo Tajima, Aichi Cancer Center Research Institute.
